# Old cells, new tricks: chromatin structure in senescence

**DOI:** 10.1007/s00335-016-9628-9

**Published:** 2016-03-28

**Authors:** Aled John Parry, Masashi Narita

**Affiliations:** Li Ka Shing Centre, Cancer Research UK Cambridge Institute, Robinson Way, Cambridge, CB2 0RE UK

## Abstract

Cellular senescence is a stable form of cell cycle arrest with roles in many pathophysiological processes including development, tissue repair, cancer, and aging. Senescence does not represent a single entity but rather a heterogeneous phenotype that depends on the trigger and cell type of origin. Such heterogeneous features include alterations to chromatin structure and epigenetic states. New technologies are beginning to unravel the distinct mechanisms regulating chromatin structure during senescence. Here, we describe the multiple levels of chromatin organization associated with senescence: global and focal, linear, and higher order.

## Introduction

More than 50 years ago Hayflick and Moorhead formally described cellular senescence as the stable proliferative arrest observed in primary human fibroblasts when cultured in vitro for extended periods (Hayflick and Moorhead 1961; Hayflick 1965). Today, we know that the phenomenon they observed represents one type of senescence, replicative senescence, which is triggered because dividing cells, in the absence of telomerase, progressively shorten their telomeres. Critically short telomeres malfunction and trigger senescence through the DNA damage response (DDR) (Shay and Wright 2000; Von Zglinicki et al. 2005). Similar senescence phenotypes can be triggered by various pathophysiological stimuli including stress from cytotoxic/genotoxic drugs, modulation of chromatin states (Ogryzko et al. 1996; Prieur et al. 2011), high levels of reactive-oxygen species (ROS) (Chen et al. 1995; Lee et al. 1999; Macip et al. 2002), and hyper-activation of oncogenes such as RAS, causing oncogene-induced senescence (OIS) (Serrano et al. 1997; Gorgoulis and Halazonetis 2010). Although originally defined in cell culture models, senescence has also been identified in vivo. For example, senescence plays a functional role in tissue homeostasis and wound healing (Wiemann et al. 2002; Krizhanovsky et al. 2008; Jun and Lau 2010), embryonic development (Storer et al. 2013; Chuprin et al. 2013; Muñoz-Espín et al. 2013), aging, and age-related disorders including cancer, atherosclerosis, and osteoarthritis (Minamino et al. 2002; Price et al. 2002; Muñoz-Espín and Serrano 2014; Pérez-Mancera et al. 2014).

**Fig. 1:**
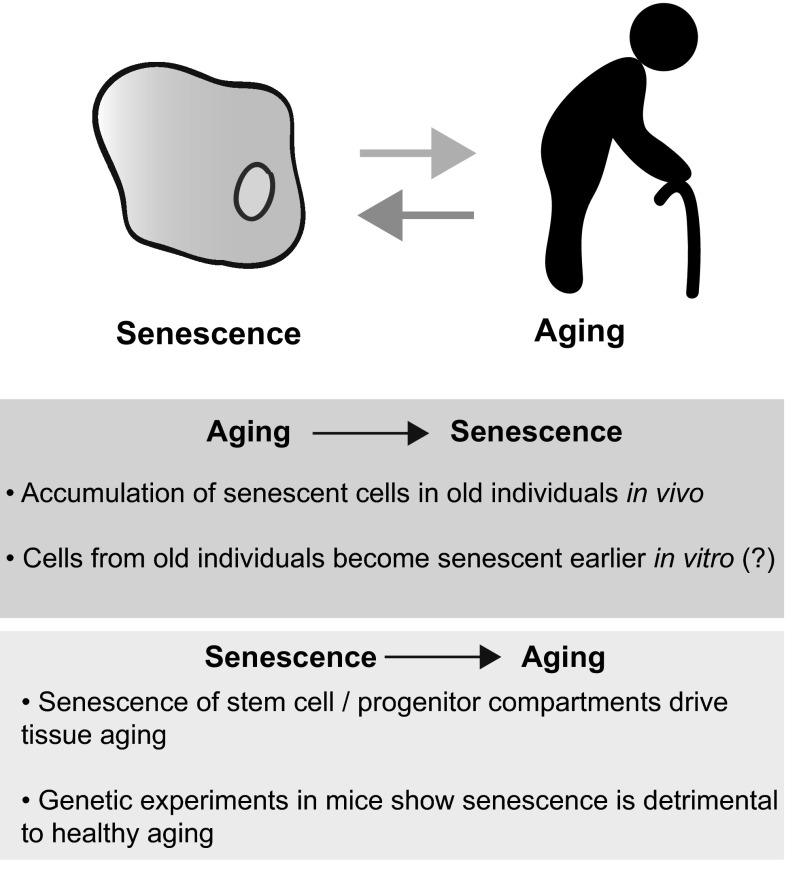
Cellular senescence and organismal aging. Although these processes involve different levels of complexity, evidence linking senescence and aging has been accumulating. Correlation between aging and senescence (*Aging* >Senescence), and some functional relevance of senescence for the aging process (*Senescence* >Aging) are shown. (*Question mark*) represents that the statement appears to be controversial (see text)

Since originally suggested in Hayflick’s seminal description (Hayflick and Moorhead 1961), evidence linking senescence and aging has been accumulating (note here that we use the term ‘senescence’ as the cell phenotype described above, distinct from ‘aging,’ a term we use in reference to organismal aging) (Fig. 1). Earlier studies showed an inverse correlation between proliferative capacity of cells in culture and donor age, although this appears to be controversial (Cristofalo et al. 2004). Senescent cells have been shown to accumulate in some (but not all) tissues of aging mice and primates (Herbig et al. 2006; Jeyapalan et al. 2007; Wang et al. 2009). In particular, age-dependent accumulation of senescent cells in tissue stem and progenitor cell compartments suggests that senescence could contribute to aging by limiting the regenerative capacity of the tissue (reviewed in Sharpless and DePinho 2007). Consecutive studies in BubR1 mice (Baker et al. 2008, 2011), a progeroid model that has high levels of constitutive DNA damage due to a hypomorphic mutation in *Bub1b*, and more recently in naturally aged mice (Baker et al. 2016), suggest that an accumulation of senescent cells has a detrimental effect on longevity (Kapanidou et al. 2015). Here, genetic inactivation of senescence or the induction of apoptosis in senescent cells (specifically p16-expressing cells) significantly attenuates the progression of age-related disorders (Baker et al. 2008, 2011, 2016). Thus understanding the diverse phenotype referred to as ‘senescence,’ and its functional roles in vivo, would likely provide insights into the various aspects of organismal aging.

Senescence involves various effector mechanisms including the DDR, alterations to cellular metabolism and protein secretion as part of the senescence-associated secretory phenotype. These senescence effectors are extensively reviewed elsewhere (Salama et al. 2014; Muñoz-Espín and Serrano 2014; Pérez-Mancera et al. 2014). In this Review, we focus on the dramatic higher-order chromatin structural changes observed during senescence, including a rearrangement of heterochromatin, and the effectors responsible. First, we discuss general aspects of heterochromatin assembly and what is known about heterochromatin in senescent and aging-associated cells. We comment on the potential implications of chromatin structure in senescent cells on gene regulation and age-related disease initiation, particularly cancer. Finally, we describe higher-order chromatin structure in senescent cells and discuss how recent research suggests that distinct regulatory mechanisms are at work during senescence.

## Chromatin

Chromatin constitutes one of the most complex macromolecular entities in the cell, harboring genomic DNA as well as thousands of directly or indirectly associating proteins and RNA molecules working in concert to mediate gene transcription or repression. Fitting human DNA within the relatively tiny boundaries of the nucleus requires several magnitudes of coiling and compaction. Around 146 base pairs of DNA are coiled around a nucleosome; a fundamental protein complex composed of an octamer of paired histone proteins—H2A, H2B, H3, and H4. Unstructured N- and C-terminal histone tails protrude from the nucleosome complex susceptible to decoration with a variety of post-translational modifications (PTMs, often called *histonemarks*). These modifications can have profound effects on chromatin structure and can therefore demarcate active and repressed domains (Luger et al. 2012).

The packaging of chromatin is highly dynamic; it can be loosely packaged euchromatin and thus allow access to the transcriptional machinery, or tightly packaged heterochromatin thereby limiting access. Heterochromatin can be further classified into two subtypes—constitutive heterochromatin is condensed in all differentiated cell types throughout the cell cycle. Facultative heterochromatin, the second subtype, is found at developmentally regulated loci and therefore varies between cell types and in response to cellular signals.

Emil Heitz (1928) originally defined heterochromatin based on the dense pattern of DNA staining detectable via microscopy of cells in interphase. Particularly, constitutive heterochromatin can be observed at the nuclear periphery and in the telomeric and pericentromeric regions of chromosomes. Since then various biochemical markers of heterochromatin and/or gene repression have been characterized: for example, DNA methylation (specifically 5-methylcytosine in CpG dinucleotides), lysine hypoacetylation of histones H3 and H4, methylation of histone H3 on lysine 9 (H3K9me3) (enriched in constitutive heterochromatin), and H3K27me3 (enriched in facultative heterochromatin) (Grewal and Jia 2007; Moazed 2001; Trojer and Reinberg 2007). Consistently, condensed regions tend to be more resistant to DNase I digestion (Sperling et al. 1985) indicating that nucleosomes are tightly packed.

## Heterochromatin assembly

**Fig. 2:**
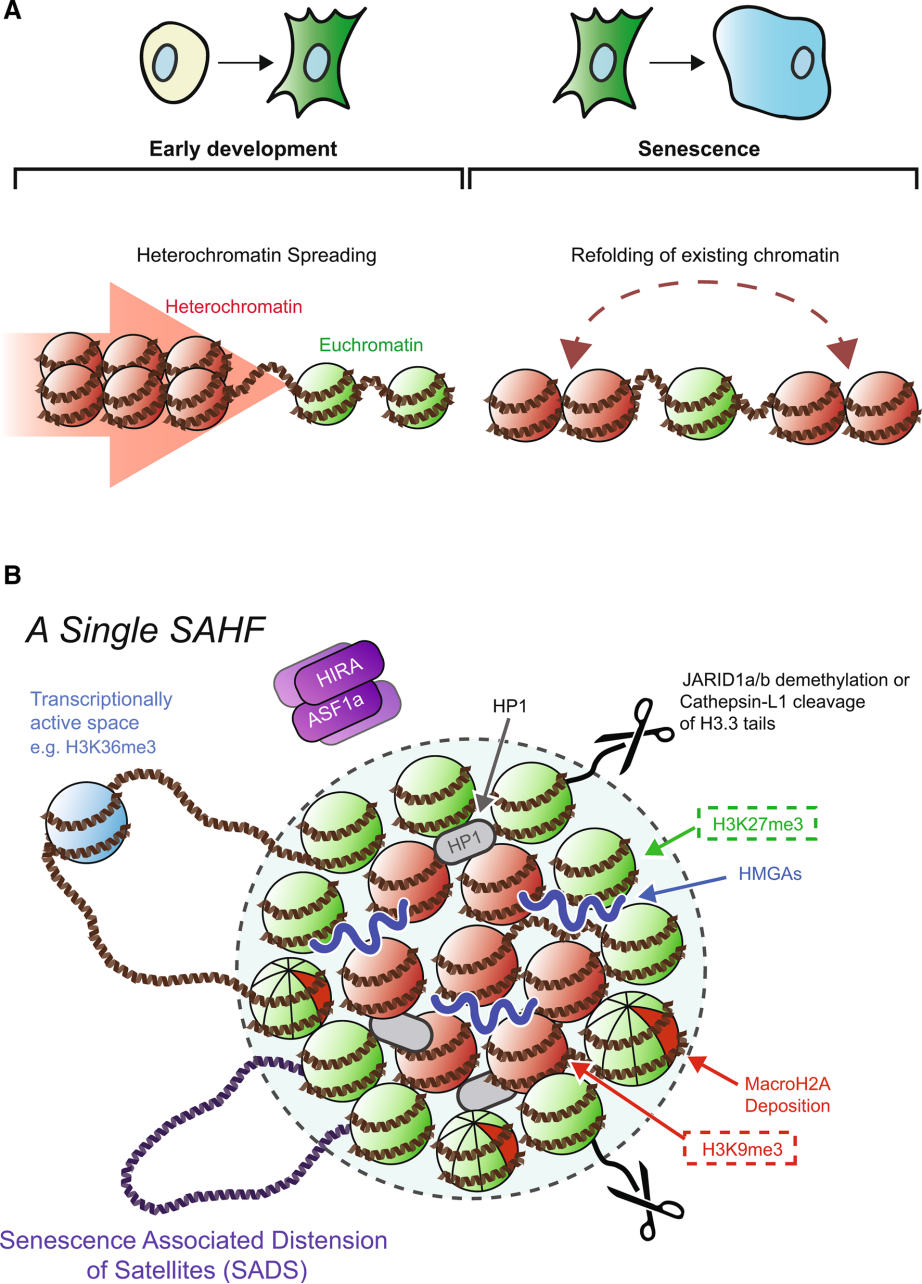
Heterochromatin assembly through spreading and spatial reorganization. **a** The primary model of heterochromatin establishment involves ‘spreading’ across the linear genome. This has largely been demonstrated in models of early development (*left*). In contrast, recent data suggest that during senescence there is a refolding of existing heterochromatin rather than an expansion (*right*). **b** Characterization of single SAHF. Chromatin is reorganized into a layered structure composing of a core enriched for the histone mark H3K9me3 (*red* nucleosomes) and an outer ring of H3K27me3 (*green* nucleosomes). Various effectors and structural components of SAHF have been identified, including HIRA/ASF1a, JARID1a/b, Cathepsin-L1, HP1 proteins, HMGA proteins, and others. Transcriptionally active genes and SADS may loop out from the SAHF core (Color figure online)

The predominant model of heterochromatin formation involves a ‘spreading’ of heterochromatin across the linear genome to proximal regions (Fig. 2a). Position-effect variegation (PEV) was first characterized by Muller (Muller et al. 1935) in drosophila models where a euchromatic section of chromatin can be silenced if artificially juxtaposed adjacent to a segment of heterochromatin via chromosome rearrangement. This process of silencing appears to begin early on during embryogenesis (Karpen 1994; Lu et al. 1996; Ebert et al. 2006). Similarly, during initiation of random X-chromosome inactivation in the embryonic cells of female mammals, the establishment of H3K27me3 and the recruitment of polycomb group (PcG) binding proteins are early events which are closely parallel with *Xist* RNA accumulation. Such marks are thought to allow propagation of the inactive state along the chromosome (Heard et al. 2001; Mermoud et al. 2002; Silva et al. 2003; Plath et al. 2003). In addition, the spreading of repressive histone marks has been shown at a genome-wide level. ChIP-seq analyses revealed that sparse blocks of repressive H3K9me3 and H3K27me3 observed in human embryonic stem cells (ESCs) significantly expand as cells differentiate (Mikkelsen et al. 2007; Hawkins et al. 2010).

ESCs are unique in their plasticity, pluripotency, and ability to self renew. Consistently, chromatin in ESCs is globally de-condensed and enriched for euchromatic modifications (Arney and Fisher 2004; Meshorer and Misteli 2006), while heterochromatin is dispersed and poorly defined (Ahmed et al. 2010). The openness of chromatin is correlated with high DNAse I accessibility and hyper-transcription of coding genes as well as repetitive elements—e.g., satellite repeat elements (Meshorer et al. 2006; Efroni et al. 2008; Fisher and Fisher 2011). As cells differentiate and a specific transcriptional program is selected there is an accumulation of heterochromatin and silencing histone modifications (Meshorer et al. 2006; Meshorer and Misteli 2006).

Of note, beside unicellular organisms, such as yeast (Grewal and Jia 2007), the experimental models to describe heterochromatin spreading primarily involve embryonic development, which often accompanies morphological alterations to chromatin (e.g., Xi formation and ESC differentiation, discussed above). These studies suggest some correlation between heterochromatin spreading and DNA ‘compactness,’ at least during early developmental stages, but a causative role for ‘quantity’ of repressive histone marks in high-order heterochromatin formation is not entirely clear (discussed below).

## Chromatin, senescence, and aging

Alterations to epigenetic and chromatin states during senescence have long been observed. Earlier studies showed that global DNA methylation is reduced in replicatively senescent, but not in immortalized, fibroblasts from rodents and humans (Wilson and Jones 1983; Gray et al. 1991). In addition, treatment of human fibroblasts with chromatin-modifying reagents that can counter heterochromatin formation, such as inhibitors of histone deacetylases or DNA methylation, reduces their proliferative lifespan (Gray et al. 1991; Ogryzko et al. 1996). These studies have provoked the “loss of heterochromatin” model of senescence, which proposes that heterochromatic domains established during early development are broken down resulting in de-repression of silenced genes and aberrant gene expression, which may contribute to the senescence and/or aging phenotype (Villeponteau 1997). Interestingly, it has been shown that hypo-methylation at some pericentric satellite regions (constitutive heterochromatin) during replicative senescence is correlated with de-repression of transcription at these regions (Suzuki et al. 2002; Enukashvily et al. 2007; Cruickshanks et al. 2013).

More recent studies, however, suggest that the loss of heterochromatin model is too simplistic (Schellenberg et al. 2011; Cruickshanks et al. 2013). Using whole genome single-nucleotide bisulfite-sequencing, Cruickshanks and colleagues illustrated that, in human fibroblasts approaching replicative senescence, there is simultaneous global DNA hypo-methylation and focal hyper-methylation (Cruickshanks et al. 2013). DNA hypo-methylation is enriched at gene-poor, late replicating sequences, and in nuclear lamina-associated domains (LADs), which are all typically associated with heterochromatic histone marks. On the other hand, focal hyper-methylation is enriched at CpG islands, localized at gene promoters, where methylation usually results in repression of the gene. It is conceivable that the global loss of DNA methylation (and de-repression of some satellite transcripts mentioned above) might contribute to subsequent genomic instability, whereas gain of DNA methylation at CpG islands might affect cell phenotype by altering the gene expression profile. Although caution is required when directly inferring pathology of organismal aging from a replicative senescence phenotype in vitro (Cristofalo et al. 2004), given that these methylation changes are reminiscent of cancer the authors speculate that senescence-associated DNA methylation alterations might promote cancer as an age-related disorder in vivo (Cruickshanks et al. 2013). Indeed, such genome-wide hypo-methylation and focal hyper-methylation of CpG islands has also been noted in the context of aging (Reviewed in Zampieri et al. 2015).

Levels of heterochromatic histone marks have also been reported to alter during senescence, but the directionality of the change appears to be dependent on the type of senescence. For example, it was reported that the total level of H3K9me3 in OIS cells, but not replicatively senescent cells, is increased when compared to proliferative control cells in culture (Di Micco et al. 2011). Comparatively, H3K9me3, the HP1 proteins and H3K27me3 were all found to be reduced in cultured fibroblasts derived from patients with the premature aging disorder Hutchinson–Gilford progeria syndrome (HGPS), caused by a point mutation (1824 C– >T) in the *LMNA* gene, as well as in fibroblasts from old individuals (Scaffidi and Misteli 2006; Shumaker et al. 2006; Dechat et al. 2008). Of note, this reduction of heterochromatic marks appears to be particularly relevant after substantial passage in culture, thus it is not clear whether the reduction of these repressive marks are primary changes associated with individual aging or a part of a ‘senescence’ phenotype caused by replicative and oxidative stress. Indeed, HGPS cells have been shown to be more susceptible to premature senescence than unaffected control cells in culture (Liu and Zhou 2008). Interestingly, a recent study showed that the H3K9me3 level in HGPS cells is often higher at an early passage compared to cells isolated from healthy individuals (Liu et al. 2013). Similar results were obtained in embryonic fibroblasts from progeroid mice, which lack the prelamin-A processing metalloprotease, Zmpste24 (Liu et al. 2013). The authors suggest that the loss of H3K9me3 in HGPS cells observed in earlier studies might be due to the secondary effect of extensive replicative stress in culture (Liu et al. 2013). Whether or not such a rapid reduction of heterochromatin at late passage is unique to cells isolated from HGPS patients or older individuals is an interesting question, particularly considering that the global alteration of heterochromatic marks appears to be non-uniform within various types of senescence. Nevertheless, the data in this study (Liu et al. 2013) do not exclude the possibility that similar epigenetic alterations observed in late passage HGPS in culture might occur in certain types of cells (e.g., tissue stem/progenitor cells) in vivo, contributing to deterioration of the tissue microenvironment during the (premature) aging process.

Thus the enrichment of heterochromatin modifications does indeed change during senescence but directionality is difficult to dissect due to the vast heterogeneity of the phenotype. In addition, as shown in the case of DNA methylation, it will be critical to consider that individual epigenetic marks may be ‘redistributed’ at specific loci during senescence in addition to changing at a global level. Moreover and as mentioned in previous sections of this review, the link between the ‘quantity’ of heterochromatic histone marks and higher-order chromatin structure, remains unclear. A number of recent studies using new technologies have begun to study alterations to higher-order chromatin structure in senescent cells in unprecedented detail—these will be discussed in the following sections.

## Senescence-associated heterochromatic foci

Senescent cells exhibit dramatic alterations to higher-order chromatin structure. Notably, in some senescent cells there is the formation of senescence-associated heterochromatic foci (SAHFs) (Narita et al. 2003; Zhang et al. 2005), DAPI-dense foci inside the nucleus readily visible by fluorescence microscopy. It has been suggested that these structures contribute to the irreversibility of the senescence phenotype by packaging proliferative genes, such as those silenced by the retinoblastoma (RB) tumor suppressor protein, into repressed heterochromatic domains (Narita et al. 2003; Beausejour 2003; Chicas et al. 2010; Sadaie et al. 2013).

Diverse effectors of SAHF structure have been identified (Fig. 2b) (Adams 2007; Salama et al. 2014). For example, knockdown of RB and its upstream activator p16 inhibit SAHF formation (Narita et al. 2003). Studies in the Adam’s laboratory identified other key modulators of SAHF including histone chaperone proteins Histone repressor A (HIRA) and Anti-silencing Factor 1a (ASF1a) (Zhang et al. 2005, 2007). These are important for euchromatic deposition of the histone variant H3.3 during senescence (Corpet et al. 2014), where histone H3.3 can be actively demethylated at lysine 4 or proteolytically cleaved (Chicas et al. 2012; Duarte et al. 2014). Promyelocytic leukemia (PML) nuclear bodies (NBs), which become prominent in number and size during senescence (Pearson et al. 2000; Ferbeyre et al. 2000; Bischof et al. 2002), are thought to provide a convergence point for multiple factors and complexes prior to SAHF formation (e.g., Rb-E2F complexes, HP1, the HIRA/ASF1a complex, histone H3.3, and the RB phosphatase PP1α) (Zhang et al. 2005; Vernier et al. 2011; Corpet et al. 2014). The relocalization of HIRA to PML bodies was reported to be driven by repression of canonical Wnt signaling, facilitating SAHF formation (Ye et al. 2007). Another recent study from the Zhang laboratory identified SPOP, an E3 ubiquitin ligase adaptor, as a potential uprestream regulator of SAHF formation: upregulation of SPOP during senescence induces degradation of the SENP7 deSUMOylase, resulting in HP1α sumoylation. The authors suggest that increased HP1α sumoylation facilitates HP1α deposition into SAHFs, whereas it only weakly promotes its relocation to PML bodies (Zhu et al. 2015). High-mobility group A (HMGA) proteins are chromatin architectural proteins that bind the minor-groove of AT-rich DNA, especially in DNA linker-regions between nucleosomes (Reeves 2001). Upregulation of HMGA proteins and downregulation of their competitor for linker DNA, Histone-H1, were implicated in SAHF formation (Narita et al. 2006; Funayama et al. 2006). HMGA proteins in particular are essential structural components of SAHFs.

SAHFs were named as such because they are enriched for many markers of heterochromatin including HP1 proteins, the facultative heterochromatin histone variant macroH2A, H3K9me3, and H3K27me3, while they exclude euchromatic markers such as H3K4me3 and H3K36me3 (Narita et al. 2003; Zhang et al. 2005). Interestingly, it has been shown that individual SAHFs are composed of single chromosomes (Funayama et al. 2006; Zhang et al. 2007). Furthermore, previous studies in our lab have shown that SAHFs are made up of multiple forms of chromatin arranged into concentric layers. Each consists of a core enriched for constitutive heterochromatin marked by H3K9me3 encircled by facultative heterochromatin enriched for H3K27me3. Euchromatic histone marks lie outside of the SAHF structure in DAPI-poor regions that probably represent transcriptionally active space (Chandra et al. 2012).

Despite the dynamic structural alteration of chromatin (i.e., SAHF formation), it was shown that the ‘chromosome-wide’ profiles of two representative repressive marks, H3K9me3 and H3K27me3, as determined by ChIP-seq, change only moderately (with some local enrichment of H3K27me3 in gene-centric analyses) upon the induction of OIS in human fibroblasts even though the total level of chromatin bound HP1, which docks at H3K9me3, is substantially increased (Chandra et al. 2012; Salama et al. 2014). These data suggest that SAHFs are not generated by a substantial re-distribution, or ‘spreading,’ of histone modifications across the genome, but rather by a spatial rearrangement of pre-existing heterochromatin involving a clustering of regions which share specific histone modifications (Chandra et al. 2012; Chandra and Narita 2013).

How can such a drastic mobilization of heterochromatin be achieved to form SAHFs? There is now a wealth of literature illustrating senescence-associated alteration to the nuclear lamina, major components of which include A-type and B-type Lamins, which form a scaffold underneath the inner nuclear membrane. Lamin B1 in particular is down-regulated during senescence (Shimi et al. 2011; Freund et al. 2012; Dreesen et al. 2013; Shah et al. 2013; Sadaie et al. 2013) through diverse mechanisms that involve transcriptional and/or post-translational regulation, the latter including the macromolecule degradation machinery, autophagy (Ivanov et al. 2013; Lenain et al. 2015; Dou et al. 2015). The nuclear lamina has been implicated in the nuclear positioning of chromatin and transcriptional regulation. Genome-wide mapping of Lamin B1 identified (sub)megabase-sized, well-defined chromatin domains (LADs), which are enriched for repressive marks (Guelen et al. 2008). Consistent with the primarily perinuclear localization of H3K9me3 in normal human fibroblasts, ChIP-seq analyses suggest that H3K9me3-enriched chromatin domains are typically associated with the middle of LADs. Interestingly, Lamin B1 is reduced mostly from the central, H3K9me3-enriched, portion of LADs during senescence (Sadaie et al. 2013). These data suggest that the preferential loss of Lamin B1 from H3K9me3-enriched regions facilitates the spatial reorganization of constitutive heterochromatin into SAHFs, although enforced depletion of Lamin B1 is not sufficient for SAHF formation (Sadaie et al. 2013). Interestingly, it has been shown that, upon differentiation of rod cells in the eyes of nocturnal mammals, a co-absence of LBR (Lamin B1 receptor), which can bind HP1 (Ye and Worman 1996, Ye et al. 1997), and lamin A/C facilitates the internalization of constitutive heterochromatin (Solovei et al. 2009, 2013). These data may suggest the existence of an evolutionarily conserved mechanism for spatial regulation of heterochromatin.

Although the correlation between histone marks and chromatin layering in SAHFs is strong, we have previously shown that depletion of H3K9me3 or H3K27me3, by ectopic expression of the histone demethylase JMJD2D or by RNAi depletion of the essential component in the histone methyltransferase complex SUZ12, respectively, does not affect SAHF formation, at least at a morphological level (Chandra et al. 2012). Moreover, others have shown that marked depletion of chromatin bound HP1 proteins by overexpression of a dominant-negative HP1β does not cause a defect in SAHF formation (Zhang et al. 2007), suggesting that the non-stoichiometric increase of these proteins when compared to their binding site is not responsible for SAHF structure. Therefore, although correlated with the layers of SAHF, these abundant markers of heterochromatin are not required for SAHF formation.

Such observations were perhaps surprising. They are in stark contrast to the predominant model of heterochromatin formation observed during embryonic development, which involves the spreading of heterochromatic marks to proximal regions. It appears that distinct regulatory mechanisms are employed in senescent cells when compared to differentiating cells at early embryonic stages: *refolding of higher*-*order chromatin structure* above the level of nucleosome modification versus *spreading of heterochromatin*, respectively (Fig. 2a), although these may not necessarily be mutually exclusive.

## Senescence-associated distension of satellites

It was recently shown by Swanson and colleagues that normally compacted α-Satellite and Sat-II DNA are distended early during the senescence program, an observation they called Senescence-associated distension of satellites (SADS) (Swanson et al. 2013). While SAHFs occur only in some forms of senescence (Narita et al. 2003; Kosar et al. 2011) and are rarely observed in vivo (Lazzerini Denchi et al. 2005), the authors observed the presence of SADS in many senescent cell types including in OIS and replicative senescence as well as in tissue sections of benign pancreatic intraepithelial neoplasia (PIN). SADS were also present in HGPS cells, which do not exhibit SAHF (Swanson et al. 2013). Moreover, they showed that the distribution of H3K9/27me3 across α-Satellite DNA does not change during senescence—further illustrating higher-order chromatin structural alterations above the level of nucleosome modification during senescence.

Evidence for an alteration to nucleosome density during replicative senescence was provided in a study carried out by the lab of John Sevidy (De Cecco et al. 2013). Researchers used a biochemical approach: formaldehyde-assisted isolation of regulatory elements (FAIRE-Seq), a method for mapping the distribution of nucleosome-depleted chromatin in comparison to nucleosome dense chromatin irrespective of histone modification (Giresi et al. 2007). They observed a general ‘smoothening’ of the genome architecture such that regions that are usually heavily heterochromatinized with respect to nucleosome positioning, including pericentromeric constitutive heterochromatin and repeat elements, became relatively more open, while open regions, with the exception of some specific genes, became relatively more closed (De Cecco et al. 2013). Particularly, and consistent with the formation of SADS, it was shown that the transcription of satellite DNA as well as transposable elements is increased (De Cecco et al. 2013). This is reminiscent of DNA methylation alterations during replicative senescence as described earlier: the global reduction of methylation, including pericentric heterochromatic regions, and focal increase of DNA methylation at CpG islands, which are generally open (Cruickshanks et al. 2013). The DNA methylation profile of OIS cells has not yet been determined and the relationship between DNA methylation, heterochromatic histone marks, and nucleosome position/density in this context remains to be elucidated.

**Fig. 3:**
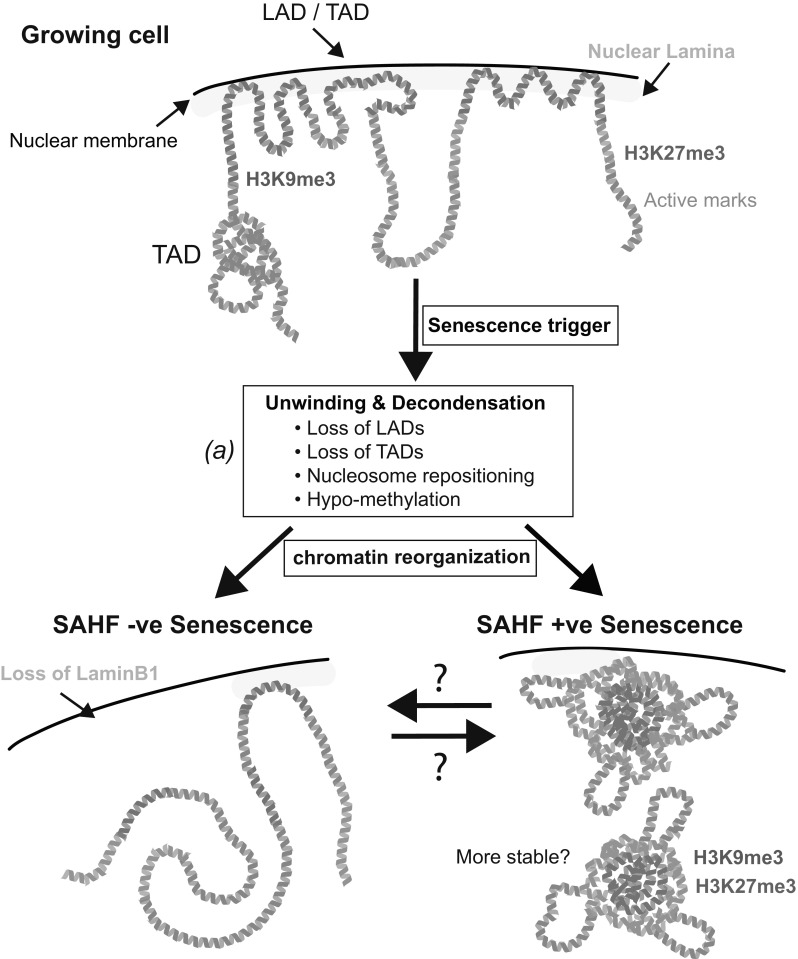
A speculative model of high-order chromatin structure alterations during senescence. Recent data suggest a ‘modular’ rearrangement of chromatin during senescence—an unwinding of chromatin and a de-condensation of canonical heterochromatin, its spatial reorganization and a refolding of chromatin. (*a*) It is unknown how general the features of unwinding are (i.e., loss of LADs, TADs etc.) and the extent of these may vary between types of senescence. Here, we illustrate two possible states following refolding (SAHF −ve and +ve), although a spectrum of states is likely. Although we have depicted chromatin as linear in the SAHF −ve state, various higher-order structures may exist

With all these data in mind, it would also be tempting to hypothesize that during senescence there is a ‘modular’ process of structural rearrangement—an unwinding of chromatin and a de-condensation of canonical heterochromatin, its spatial reorganization, and a refolding of chromatin to form the layered structure of SAHF (Fig. 3).

## The genome-wide chromatin interactome

Historically, the study of higher-order chromatin structure genome-wide has been difficult as many high-throughput sequencing methods such as chromatin immunoprecipitation and FAIRE-Seq provide one-dimensional information about the genome. More recently, methods based on high-throughput chromatin conformation capture (3C) have allowed for the study of three-dimensional structure genome-wide (Lieberman-Aiden et al. 2009; Belton et al. 2012). This technology provided structural validation to chromosome territories by defining sub-chromosomal compartments; type A (‘open’) and B (‘closed’) compartments (Lieberman-Aiden et al. 2009), and more recently identified smaller megabase pair sized self-interacting chromatin blocks, topologically associated domains (TADs) (Dixon et al. 2012; Nora et al. 2012). Many TADs can be considered as discrete regulatory units, where a TAD may be largely heterochromatic or euchromatic as indicated by biochemical markers (Le Dily et al. 2014). Interestingly, TAD structure is conserved as ES cells differentiate into fibroblasts demonstrating that heterochromatin spreading occurs within the framework of TADs and that higher-order chromatin structure is maintained (Nora et al. 2012), even though heterochromatin at a nucleosome level changes dramatically (Mikkelsen et al. 2007; Hawkins et al. 2010). Moreover, depletion of heterochromatic modifications does not affect TADs (Nora et al. 2012).

The independent relationship between TADs and repressive histone marks is reminiscent of the relationship between SAHF formation and histone marks, which are closely correlated with the layered structure of SAHF but are not required for their formation. Are TADs a structural unit of SAHF? Recently, Chandra and colleagues (2015) employed Hi-C to understand alterations to higher-order chromatin structure during OIS. Interestingly, they observed a reduction of local connectivity in TADs that are associated with heterochromatic features, including H3K9me3 enrichment, LADs, GC-poor (AT-rich) isochores, and late replication timing. Thus, TADs, which are highly conserved structural units during differentiation and across multiples species (Dixon et al. 2012; Nora et al. 2012), are substantially modulated during OIS. Consistent with the microscopic features of SAHFs described above, they also showed that this loss of TAD structure is accompanied by an increased number of long-range chromatin interactions representing a spatial clustering of H3K9me3 domains (Chandra et al. 2015). Interestingly, cells isolated from patients with HGPS also exhibited a loss of local connectivity in TADs (Chandra et al. 2015). Since it was reported that HGPS cells are SAHF negative, it appears that the loss of TAD structure and SAHF formation are separable events. Indeed, the long-range interactions, representing A/B compartments, are drastically lost in late passage HGPS cells (McCord et al. 2013), and the spatial clustering of H3K9me3 domains observed in OIS cells appears to be lacking in HGPS cells (Chandra et al. 2015), further reinforcing a modular model where a combination of multiple processes is required for SAHF formation (Fig. 3). More recently, a Hi-C study of replicatively senescent fibroblasts, which also infrequently form SAHFs, was reported: similar to, but less intensively than, late passage HGPS cells, replicative senescence illustrated a decrease in long-range interactions; however, in contrast to the OIS study (Chandra et al. 2015), TADs are relatively conserved and short-range interactions are rather increased during replicative senescence (Criscione et al. 2016). The reason for the difference in Hi-C structure between OIS and replicative senescence remains unclear, but these studies raise important questions, such as whether the reduction of (a subset of) TADs and modulation of long-range interactions are required for SAHF formation, and how these structural alterations affect gene expression.

## Conclusions

Morphological and biochemical analyses have defined multiple levels of chromatin structure, from nucleosomes (their modifications and density) to macroscopic chromosome structures. Levels of structure between these two extremes include chromatin looping, LADs, TADs, and higher-order sub-chromosomal compartments: type A (‘open’) and B (‘closed’) (Gibcus and Dekker 2013). Researchers have now started to characterize these levels of structure in the context of senescence, and found some correlation between (the alterations of) them during senescence. These data appear to predict a modular model as discussed above; chromatin alterations during senescence involve multiple processes that affect different levels of structure, although (in)dependency between the levels is not entirely clear. This model may be consistent with the heterogeneous nature of the chromatin phenotype in different types of senescence (e.g., SAHF-positive or negative senescence), which might be in part due to different efficiencies of individual modules.

A number of factors that affect SAHF formation have been identified, as discussed above, and it will be necessary to understand which chromatin level or modular process these factors regulate during the establishment of senescence. For example, it was recently shown that condensin II is required for SAHF formation and that ectopic expression of condensin II induces SAHF formation (Yokoyama et al. 2015). Condensins, which are essential for mitotic chromosome assembly and segregation, play diverse biological roles beyond mitosis, and condensin II in particular has been implicated in the spatial organization of chromosomes during interphase (Bauer et al. 2012). In addition, condensin II is enriched at the borders of TADs, thus it would be interesting to test whether ectopically expressed condensin II contributes to senescence-associated chromatin alteration by remodeling TAD structure during senescence (Ito and Narita 2015). It will also be important to functionally link transcriptional activity and 3D chromatin structure in senescence.

These analyses will provide further insights into the various levels of chromatin and how they affect the quality of senescence, and potentially aging and age-related disorders.
